# Supplementary figures and images for: Apis andreniformis associated Actinomycetes show antimicrobial activity against black rot pathogen (Xanthomonas campestris pv. campestris)
Source: PeerJ. 2021 Sep 9;9:e12097. doi: 10.7717/peerj.12097 (PMC8435200; doi:10.7717/peerj.12097)

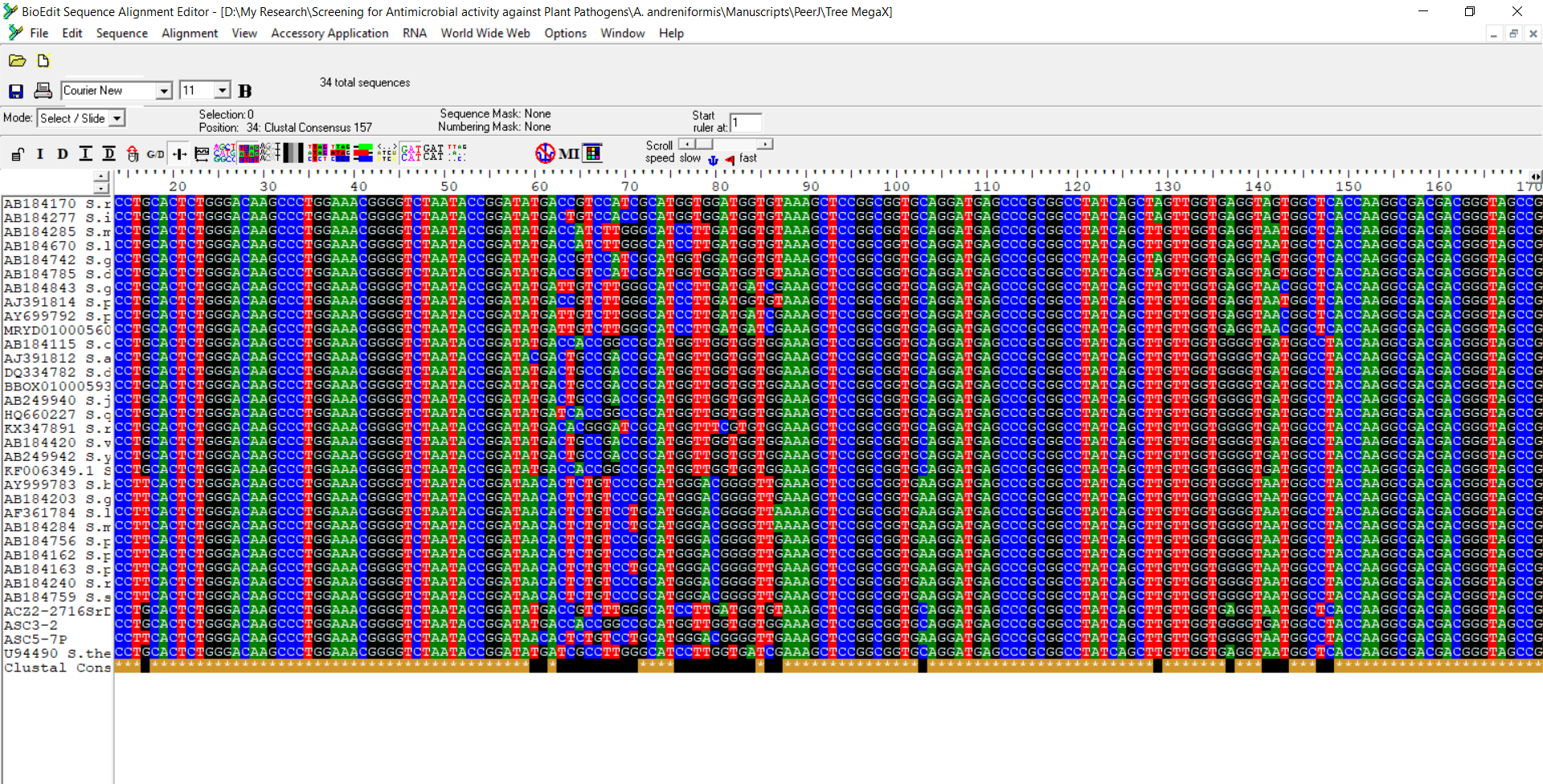

Supplement: Supplemental Information 1 [file peerj-09-12097-s001.png]

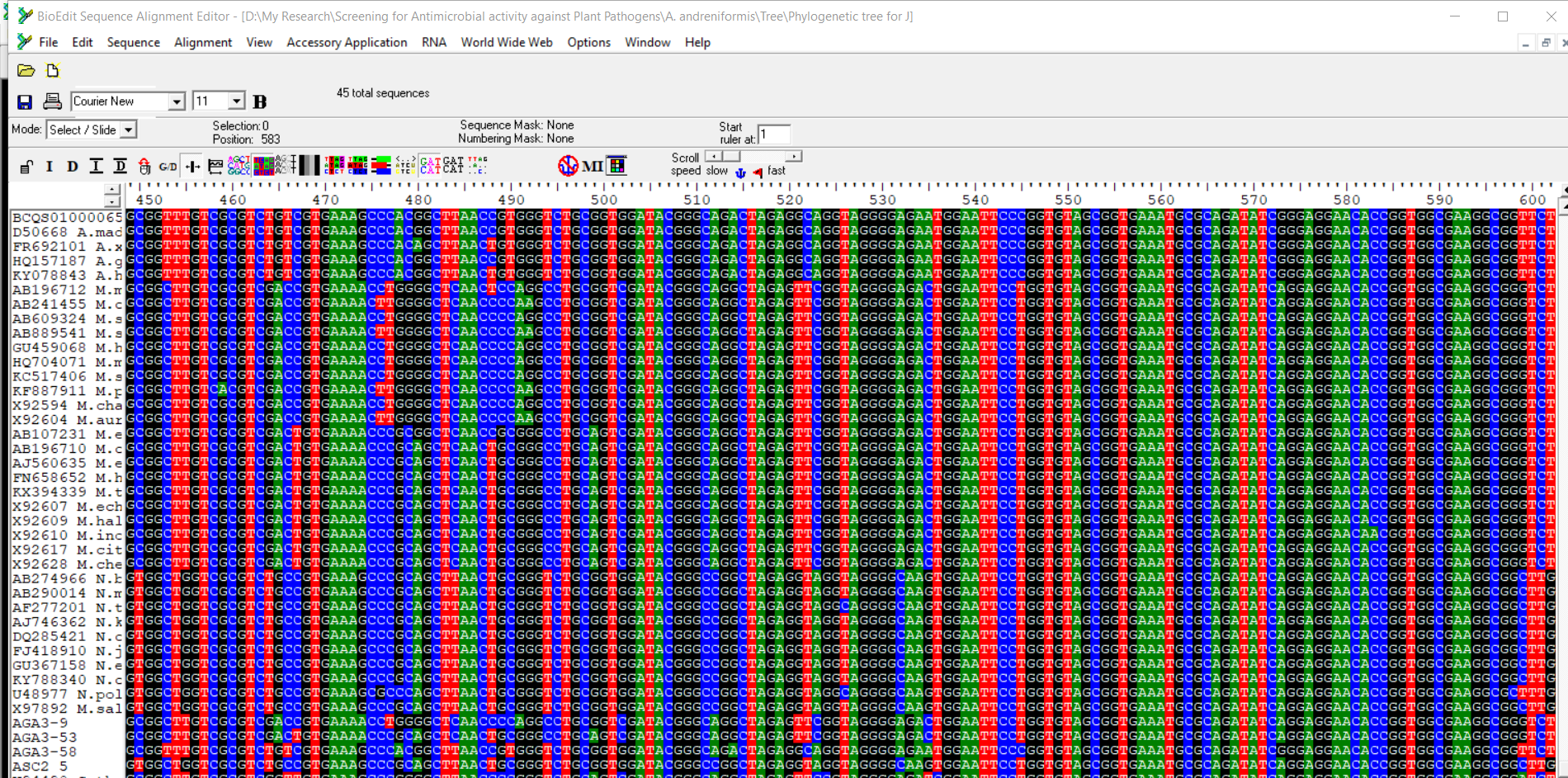

Supplement: Supplemental Information 2 [file peerj-09-12097-s002.png]
